# PlaNC-TE: a comprehensive knowledgebase of non-coding RNAs and transposable elements in plants

**DOI:** 10.1093/database/bay078

**Published:** 2018-09-13

**Authors:** Daniel Longhi Fernandes Pedro, Alan Péricles Rodrigues Lorenzetti, Douglas Silva Domingues, Alexandre Rossi Paschoal

**Affiliations:** 1 Department of Computer Science, Bioinformatics Graduation Program (PPGBIOINFO), Federal University of Technology - Paraná, Cornélio Procópio, PR, Brazil; 2 Department of Biochemistry and Immunology, Ribeirão Preto Medical School, University of São Paulo, Ribeirão Preto, SP, Brazil; 3 Department of Botany, Institute of Biosciences, São Paulo State University, UNESP, Rio Claro, SP, Brazil

## Abstract

Transposable elements (TEs) play an essential role in the genetic variability of eukaryotic species. In plants, they may comprise up to 90% of the total genome. Non-coding RNAs (ncRNAs) are known to control gene expression and regulation. Although the relationship between ncRNAs and TEs is known, obtaining the organized data for sequenced genomes is not straightforward. In this study, we describe the PlaNC-TE (http://planc-te.cp.utfpr.edu.br), a user-friendly portal harboring a knowledgebase created by integrating and analysing plant ncRNA-TE data. We identified a total of 14 350 overlaps between ncRNAs and TEs in 40 plant genomes. The database allows users to browse, search and download all ncRNA and TE data analysed. Overall, PlaNC-TE not only organizes data and provides insights about the relationship between ncRNA and TEs in plants but also helps improve genome annotation strategies. Moreover, this is the first database to provide resources to broadly investigate functions and mechanisms involving TEs and ncRNAs in plants.

## Introduction

Transposable elements (TEs) are DNA sequences capable of moving from one position to another in its own genome. They are classified into Classes, Superfamilies and Families based on structural characteristics and mechanisms of transposition (1,2). TEs in some plant species can reach about 90% of the nuclear genome, like in *Triticum aestivum* (3,5), *Gossypium raimondii* (4) and *Zea mays* (6). TEs are also recognized as a source of non-coding RNAs (ncRNAs) in genomes (7,8). ncRNAs are sequences that are not translated into proteins and have influence on great variations in biological functions (9–11). For example, they are important for gene expression regulation at post-transcriptional levels, RNA processing and translation (12). Nowadays, nearly 50 classes of non-coding elements are known (13,14). The most studied are transporter RNA (tRNA), ribosomal RNA (rRNA), microRNA (miRNA), small nuclear RNA (snRNA), small nucleolar RNA (snoRNA) and long ncRNA (lncRNA).

Previous studies show that a substantial number of previously annotated plant ncRNAs are identical or homologous to TEs. Studies performed by Piriyapongsa *et al.* (15) showed TE *loci* overlapping with 12 and 83 miRNAs in *Arabidopsis thaliana* and *Oryza sativa*, respectively. In tomato (*Solanum lycopersicum*), Wang *et al.* (16) identified 55 lncRNA genes exclusively found in this species. From these 55 species-specific lncRNAs, 47 (∼85%) overlap TEs.

Plant Transposable Element-related microRNA Database (PlanTE-MIR DB) (17), developed by our group, was the first database devoted to assembling data related to miRNAs associated with TEs, in which 152 overlapping occurrences in 10 plant genomes were described. Analyses were restricted to miRNAs and to plants whose TE annotation was available in RepBase. However, the impact of TEs in generating ncRNAs and regulating molecular processes is still a mostly studied process in vertebrates (18–21). In plants, the absence of an accurate analysis of TE-ncRNAs in sequenced genomes may explain the scarcity of studies in this area. This knowledge gap motivated us to deliver the Plant Non-Coding RNAs related to TEs (PlaNC-TE) database, which presents the results of a systematic analysis of 53 genomes available on Ensembl Plants. Moreover, we expanded PlanTE-MIR analysis to nine ncRNA classes that overlaps TEs, resulting in 14 350 ncRNA-TE occurrences in 40 species. The database portal allows users to browse, search and download all ncRNA and TE data.

**Table 1:** Types of annotated ncRNAs used in PlaNC-TE

Types of ncRNA	ncRNA classes
Long non-coding	AntisenseSense-intronic
Short non-coding	rRNAtRNAPre-miRNAsnRNAsnoRNARNase MRPSRP RNA

## Materials and Methods

### Overview

PlaNC-TE pipeline steps consist of (i) selecting reference genome sequences, (ii) obtaining ncRNA data, (iii) obtaining TEs data and (iv) identifying ncRNA-TE overlaps by comparing genomic coordinates (Figure 2). All the scripts are available at http://planc-te.cp.utfpr.edu.br/files_to_sync.zip.

### Genomic sequences

Fifty-three plant genomes were downloaded from Ensembl Plants version 38 (http://plants.ensembl.org). All genome data are detailed in Table S1.

### ncRNA data analysis

Non-coding data were obtained from the Ensembl ncRNA FASTA file (Table S1). We developed an in-house Perl script to extract from each FASTA header the seqid, source, start/end sequence, biotype, strand and attributes (geneID, name, biotype and description when available). This information was used to organize ncRNA features further converted to GFF3 format file. Main characteristics of retrieved ncRNA families are described in Tables 1 and 2.

**Table 2:** ncRNAs summary

ncRNA classes	Total
rRNA	11 226
tRNA	21 972
snRNA	6185
sense-intronic	2468
pre-miRNA	4798
snoRNA	10 759
SRP	737
antisense	176
RNase MRP	63

Those records were filtered to remove redundancies. We considered only information produced by Ensembl annotation, discarding third-party information using bash and Perl scripts. We retrieved a total of 58 390 ncRNA entries in 53 genomes.

### TEs data analysis

The information provided by Ensembl for TEs is not as organized as ncRNA data. It does not have an annotation file, so we had to gather the data from .sql files that contains repeats information (Table S1).

To extract information regarding TEs, we used the files ‘repeat_consensus’, ‘repeat_feature’ and ‘seq_region’ from each genome, available at ftp://ftp.ensemblgenomes.org/pub/release-38/plants/mysql.

We then created an in-house bash script to identify TE types and to remove non-TE data, i.e. low complexity, dust, centromeric, simple repeats, direct, artifact and ribosomal repeats. We obtained the following information from these three tables: (i) from repeat_consensus we retrieved Name, Class and Type; (ii) from repeat_feature we obtained Start/End position, Score and Strand (+/-); and (iii) from seq_region we recovered the seqID for *loci*. With this information, we executed a query using SQL syntax, which returned records exportable to a GFF3 format file.

A total of 31 217 630 TE entries were found in 45 genomes. In eight genomes (*Brassica napus*, *Cucumis sativus*, *Dioscorea rotundata*, *Helianthus annuus*, *Lupinus angustifolius*, *Manihot esculenta*, *Nicotiana attenuata* and *Trifolium pratense*), we did not obtain TE entries in Ensembl Plants, making it impossible to analyse ncRNA-TE overlaps. Finally, we also compared TEs in Repbase to our TE data set (BLASTn—version 2.6.0+, 80% identity in at least in 80 nt). Results are available in Table S2.

### ncRNA-TE overlaps

We used the intersection function from BEDTools (version 2.26.0) (22) to perform the overlap analysis, taking as input the GFF3 files we created for ncRNA and TE entries. Every overlap was considered. We manually checked results using IGV tool (Integrative Genomics Viewer; version 2.4.1) (23).

## PlaNC-TE Implementation

The system is hosted at the Universidade Tecnológica Federal do Paraná and use Debian 9 as operating system, with Apache 2 as web server, MariaDB 15.1 as database administration and PHP 5.6 as web programming language. We also used Zend Framework 2, which implements MVC (Model, View, Controller) methodology for web development to expand for any future additional functionality. On Front-End we used HTML5 (Hyper-Text Markup Language 5), CSS3 (Cascading Style Sheet 3) and JavaScript to perform dynamic functions providing a user-friendly navigation.

**Figure 1:**
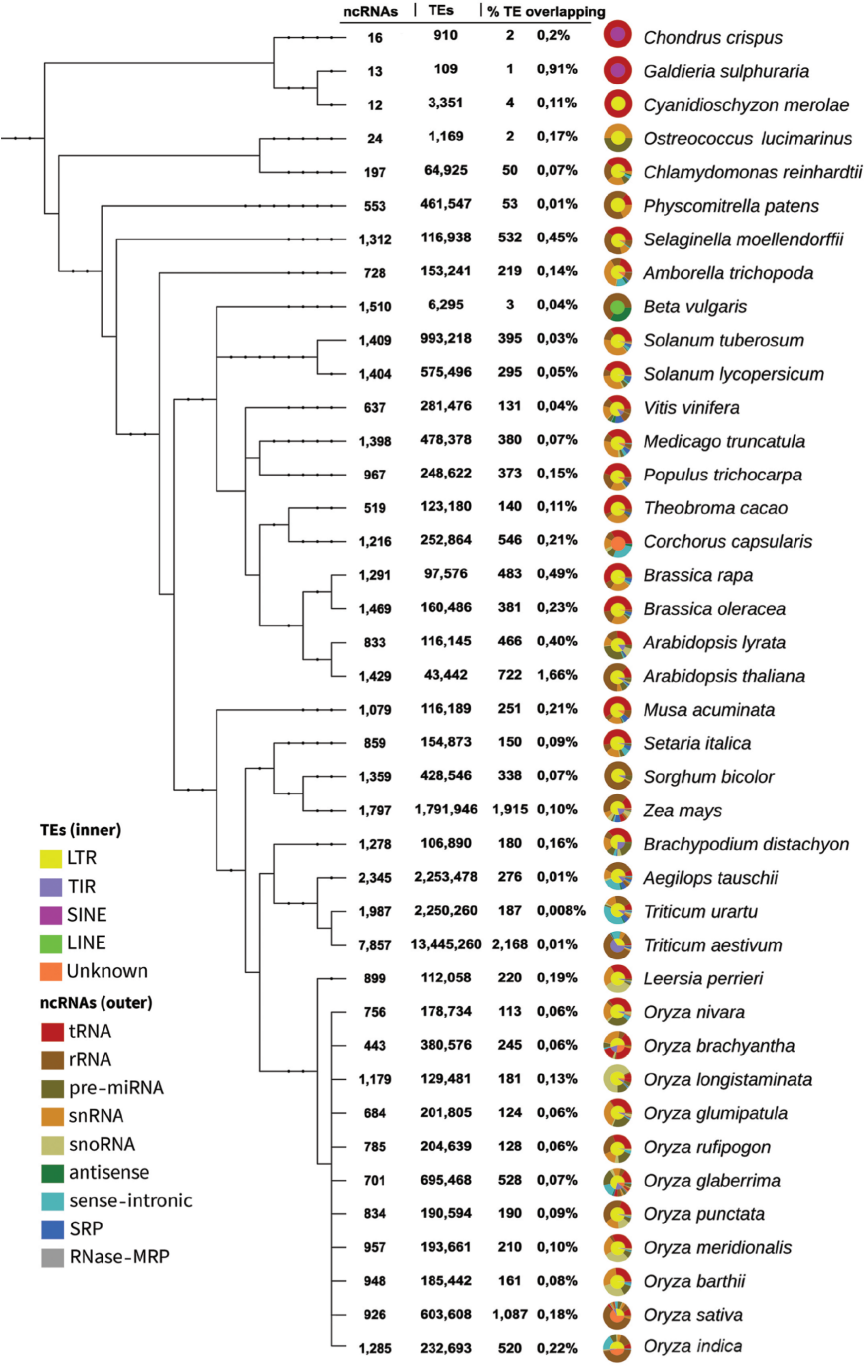
Phylogenetic tree from genomes with ncRNA-TE overlaps. The circular graphs describe the proportion of TE and ncRNA types in their inner and outer rims, respectively. This figure is also available at http://planc-te.cp.utfpr.edu.br, in which there is an interactive graphic representation.

## Results

### ncRNA-TE: an overview

**Figure 2:**
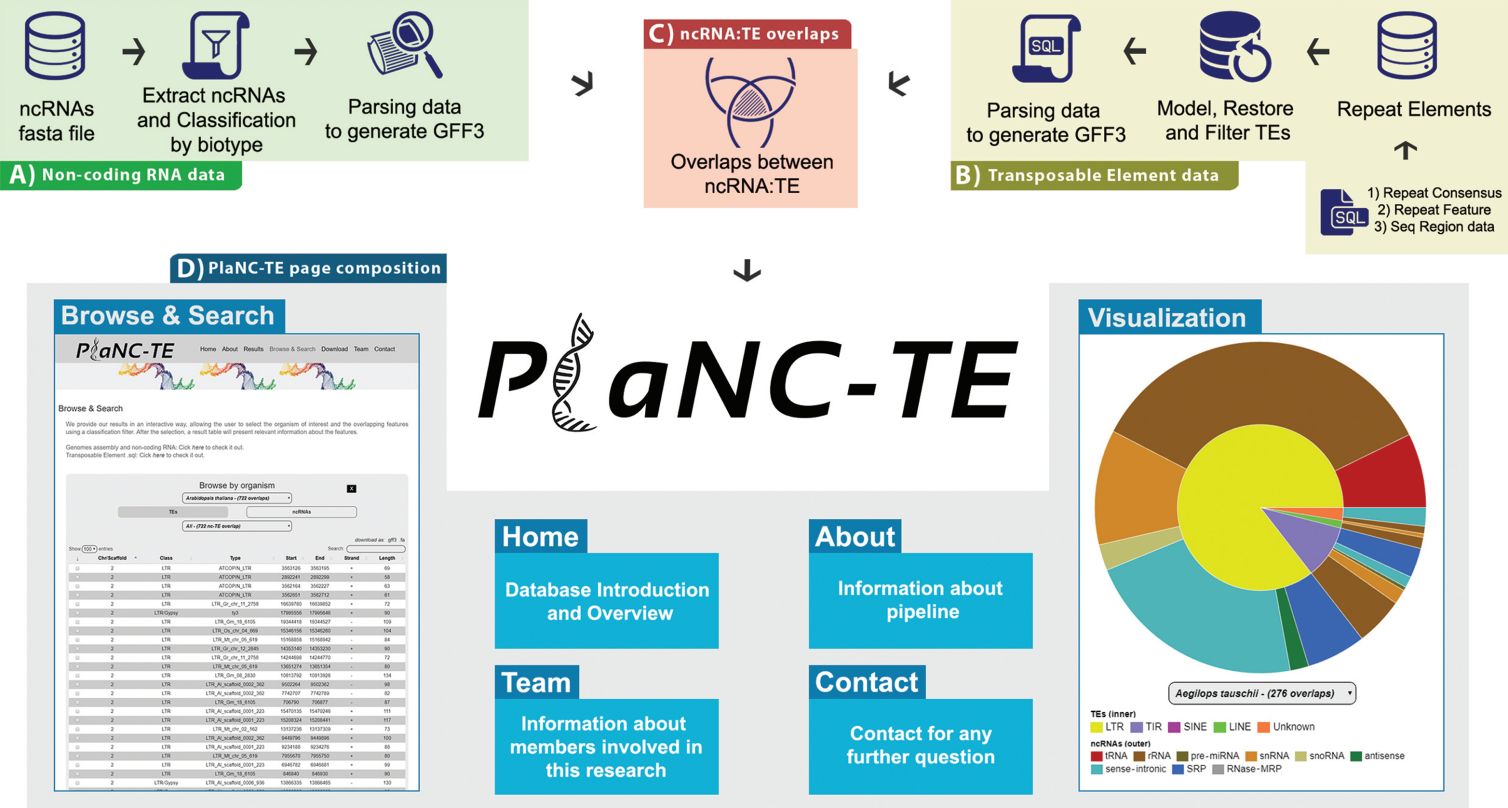
PlaNC-TE workflow: (A) ncRNA data obtention and generation of a GFF3 file. (B) Steps to obtain and filter TE data to generate a GFF3 file. (C) Overlaps between ncRNAs and TEs. (D) PlaNC-TE page composition and functionalities.

The PlaNC-TE covers all ncRNA classes and all TE types from available genomes on Ensembl Plants (Figure 2). We identified 14 350 ncRNA-TE occurrences in 40 out of 53 genomes (Figure 1 and Table 3). In five genomes (*Glycine max, Gossypium raimondii, Hordeum vulgare, Phaseolus vulgaris* and *Prunus persica*), we identified ncRNA and TE entries in Ensembl Plants, but we did not find ncRNA-TE overlaps (Table S3).

**Table 3:** Summary of ncRNA-TE overlaps

	LTR	TIR	LINE	SINE	Unknown	Total
**tRNA**	2959	192	1	14	303	3469
**rRNA**	2962	1389	25	7	1082	5465
**snRNA**	1763	117	14	2	120	2016
**Sense-intronic**	764	20	–	–	207	991
**Pre-miRNA**	696	190	3	3	94	986
**snoRNA**	529	287	2	2	49	869
**SRP**	391	70	–	–	2	463
**Antisense**	70	2	1	–	16	89
**RNase MRP**	2	–	–	–	–	2
**Total**	10 136	2 267	46	28	1873	14 350

Long Terminal Repeat (LTR); Terminal Inverted Repeat (TIR); Long Interspersed Nuclear Elements (LINE); Short Interspersed Nuclear Elements (SINE)

### ncRNA-TE characteristics

**Figure 3:**
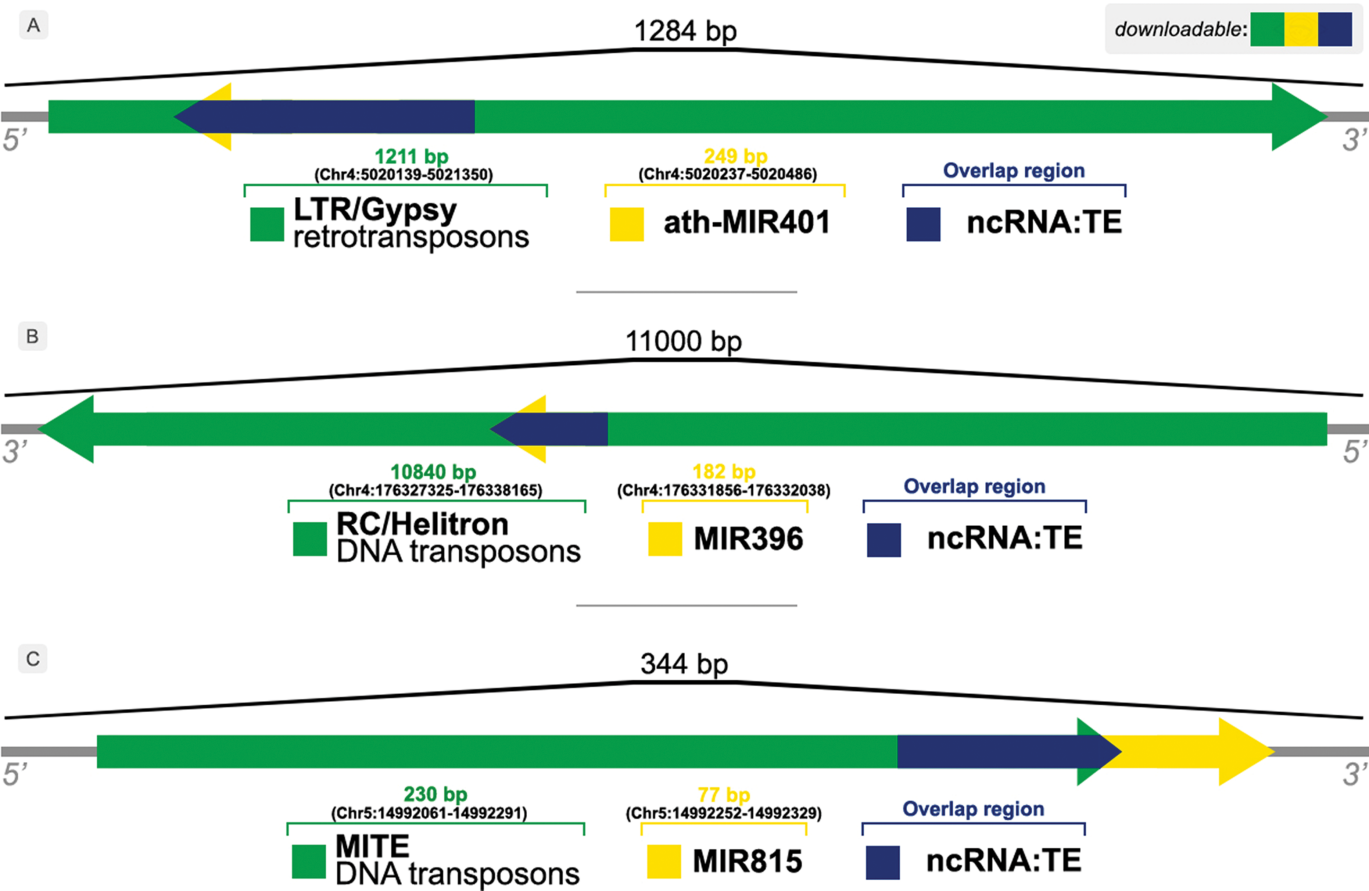
Illustration of overlaps between ncRNAs and TEs in (A) *A. thaliana*, (B) *Z. mays* and (C) *O. sativa genomes*.

The occurrence of overlaps is given by the identification of distinct element types within the same *locus/*flanking sequence, e.g. an ncRNA that has been identified inside a TE sequence, as shown in Figure 3.

We emphasize the importance of this type of analysis in a scenario characterized by the abundance of unexplored information for plant genomes, primarily considering TEs and their associated features. Several effort has been done to general annotation and organization of genomic data but few works are devoted to making sense of it.

Figure 3 shows examples of the relationship between ncRNAs and TEs found by our approach. A bulk of those overlapping sequences can be retrieved from the Download & JBrowse page on PlaNC-TE portal, but users can also retrieve sequences that overlaps each other separately using the Search interface. The files are available for download in GFF3, FASTA and TSV formats.

We noted that four genomes represent ∼41% of the total overlaps (Figure 1): (i) *Triticum aestivum* (2168), (ii) *Zea mays* (1915), (iii) *Oryza sativa* (1087) and (iv) *Arabidopsis thaliana* (722). The remainder data (∼59%) represent 36 genomes. Also, Unknown (∼13%) TE type is mostly represented by *Oryza sativa* (∼38%) and *Corchorus capsularis* (∼29%) genomes. The overlaps are distributed in percentage and related to the ncRNAs (Table 3): (i) tRNA comprises ∼24% of overlaps, in which the ratio in TEs is ∼83% for LTR, ∼7.4% for TIR, ∼0.03% for LINE, ∼0.4% for SINE and ∼8.9% for Unknown; (ii) rRNA comprises ∼38%, and overlaps are divided in ∼48% for LTR, ∼32% for TIR, ∼0.4% for LINE, ∼0.1% for SINE and ∼20% for Unknown; (iii) snRNA comprises ∼14%, overlap is divided as follows: ∼83% for LTR, ∼10% for TIR, ∼0.6% for LINE, ∼0.1% for SINE and ∼5.9% for Unknown; (iv) sense-intronic comprises ∼6.9%, with overlap with TEs divided in ∼77% for LTR, ∼2% for TIR and ∼21% for Unknown; (v) pre-miRNA comprises ∼6.8%, and overlap is divided in ∼70% for LTR, ∼19% for TIR, ∼0.3% for LINE, ∼0.3% for SINE and ∼9.5% of Unknown; (vi) snoRNA comprises ∼6%, overlap is divided as ∼60% for LTR, ∼33% for TIR, ∼0.2% for LINE, ∼0.2% for SINE and ∼5.6% for Unknown; (vii) SRP comprises ∼3.2%, overlap with TE is divided as follows: ∼70% for LTR, ∼29% for TIR, LINE and SINE no overlap were found and ∼0.4% for Unknown; (viii) antisense comprises ∼0.6%, overlap with TE is divided as follows: ∼78% for LTR, ∼2.2% for TIR, ∼1.1% for LINE, SINE no overlap were found and ∼18% for Unknown; and (ix) RNase MRP comprises ∼0.01%, overlaps with TE were exclusively with LTR elements.

### TE-miRs comparison

PlanTE-MIR DB (17) brought to the public 152 miRNA-TE overlaps for 10 plant species. Our new approach allowed PlaNC-TE DB to bring 271 miRNA-TEs (Table 4) for the same species, an increase of almost 2-fold in the number of entries. However, in *Glycine max* and *Physcomitrella patens,* no occurrences were found despite the identification of miRNA-TEs in PlanTE-MIR DB (Table 4). To uncover the issue of occurrences that was not found in PlaNC-TE, we mapped TE-MIR data from PlanTE-MIR DB in the Ensembl Plants genomes (Table S4). A total of 94% of the overlaps were maintained in another position among over the genomes. In *O. sativa*, overlaps decreased from 56 to 48 and in *S. bicolor* from 35 to 33. In *M. truncatula* and *S. tuberosum,* the overlaps increased because of duplicity on miRs and TEs, respectively.

**Table 4:** Comparison of miRNA-TE occurrences on PlanTE-MIR DB and PlaNC-TE DB

Genomes	PlanTE-MIR DB	PlaNC-TE	*Id
*A. thaliana*	22	97	21
*B. distachyon*	2	52	–
*G. max*	4	–	–
*M. truncatula*	20	19	–
*O. sativa*	56	67	2
*P. patens*	1	–	–
*P. trichocarpa*	10	3	–
*S. bicolor*	35	21	–
*S. tuberosum*	1	5	–
*V. vinifera*	1	7	–

*Id—identical overlapped records on PlanTE-MIR DB and PlaNC-TE DB.

We also directly compared the sequence of miRNA-TEs that were reported in PlanTE-MIR DB with PlaNC-TE results (Table 4, *Id column). For this, we used BLASTn (version 2.6.0+) filtering for >98% of the coverage to identify miRNA-TE correspondences. We only obtained 21 correspondences on *Arabidopsis thaliana* (21) and two on *Oryza sativa* (2).

In this work, we identified miRNAs related to TEs in 26 novel species, which result in 715 new occurrences (986 in total).

## Web Interface

PlaNC-TE (http://planc-te.cp.utfpr.edu.br) is a user-friendly web portal for the investigation of ncRNA-TEs (Figure 2) and its content is divided in seven pages.

### Home

Our main page explains the portal and also presents an interactive chart, which shows the distribution of ncRNA-TEs according to genome and features that overlap each other.

### About

It explains the analysis pipeline and provides supplementary material files.

### Reports

These are graphical representations that assist the understanding of ncRNA-TE relationship for each species.

### Search

Users can search within genomes and select which ncRNA or TE they want to download. Additionally, users can use a text field available in the right top side of the table, which lists the filtered results by keywords. Users can search and sort results by Chromosome, Class (TE), Type (TE), Class (ncRNA), Strand (+/-), Length and Overlap. The viewing mode of records can be adjusted to 10, 20, 50, 100 or All entries. Users can click and drag to select a batch of entries and download it in GFF3 or FASTA format.

### Download & JBrowse

It provides bulk files for download in GFF3 and the sequence information in the FASTA format for each species. A custom TSV file (a text file based on tab-separated values) is also available, showing information for both ncRNA and TE features involved in each overlap (Table S5). Users can also visualize ncRNAs, TEs and ncRNAs-TEs sequences in JBrowse (version 1.14.2) navigator available in PlaNC-TE portal.

### Team and contact

We also have a contact form for users to report any aspect related to PlaNC-TE DB.

## Accessibility

PlaNC-TE database is available at http://planc-te.cp.utfpr.edu.br.

## Conclusion and Future Directions

We provided comprehensive knowledge and standardized data on the relationship of ncRNAs and TEs in plants. Our results are available on a user-friendly portal allowing users to download partial or entire data.

Future versions of PlaNC-TE DB will possibly add new genomes and extend this analysis to other life domains. Also, we intend to keep PlaNC-TE DB updated with new releases of Ensembl Plants using automatic algorithms developed to perform this analysis. Finally, we plan to include in PlaNC-TE a submission interface to allow the incorporation of ncRNA-TE data generated by the scientific community.

## Supplementary Material

Supplementary Table S1

Supplementary Table S2

Supplementary Table S3

Supplementary Table S4

Supplementary Table S5

## Notes

**Database URL:** http://planc-te.cp.utfpr.edu.br/.
