# Supplementary material for: PlaNC-TE: a comprehensive knowledgebase of non-coding RNAs and transposable elements in plants
Source: Database (Oxford). 2018 Sep 13;2018:bay078. doi: 10.1093/database/bay078 (PMC6146122; doi:10.1093/database/bay078)
Supplement: Supplementary Table S1 [file bay078_tables1.pdf]

Table S1. Species, version and source for ncRNAs/TEs used by each genome.

Species	Version	Source
A. tauschii	ASM34733v1	ncRNAs<br>TEs
A. trichopoda	AMTR1.0	ncRNAs<br>TEs
A. lyrata	v.1.0	ncRNAs<br>TEs
A. thaliana	TAIR10	ncRNAs<br>TEs
B. vulgaris	RefBeet-1.2.2	ncRNAs<br>TEs
B. distachyon	v1.0	ncRNAs<br>TEs
B. oleracea	v2.1	ncRNAs<br>TEs
B. rapa	IVFCAASv1	ncRNAs<br>TEs
C. reinhardtii	v3.1	ncRNAs<br>TEs
C. crispus	ASM35022v2	ncRNAs<br>TEs
C. capsularis	CCACVL1_1.0	ncRNAs<br>TEs
C. merolae	ASM9120v1	ncRNAs<br>TEs
G. sulphuraria	ASM34128v1	ncRNAs<br>TEs
L. perrieri	Lperr_V1.4	ncRNAs<br>TEs
M. truncatula	MedtrA17_4.0	ncRNAs<br>TEs
M. acuminata	MA1	ncRNAs<br>TEs
O. barthii	O.barthii_v1	ncRNAs<br>TEs
O. brachyantha	Oryza_brachyantha.v1.4b	ncRNAs<br>TEs
O. glaberrima	AGI1.1	ncRNAs<br>TEs
O. glumaepatula	ALNU02000000	ncRNAs<br>TEs
O. indica	ASM465v1	ncRNAs<br>TEs

O. longistaminata	O_longistaminata_v1.0	ncRNAs<br>TEs
O. meridionalis	Oryza_meridionalis_v1.3	ncRNAs<br>TEs
O. nivara	AWHD00000000	ncRNAs<br>TEs
O. punctata	AVCL00000000	ncRNAs<br>TEs
O. rufipogon	OR_W1943	ncRNAs<br>TEs
O. sativa	IRGSP-1.0	ncRNAs<br>TEs
O. lucimarinus	ASM9206v1	ncRNAs<br>TEs
P. patens	ASM242v1	ncRNAs<br>TEs
P. trichocarpa	JGI2.0	ncRNAs<br>TEs
S. moellendorffii	v1.0	ncRNAs<br>TEs
S. italica	JGIv2.0	ncRNAs<br>TEs
S. lycopersicum	SL2.50	ncRNAs<br>TEs
S. tuberosum	SolTub_3.0	ncRNAs<br>TEs
S. bicolor	Sorghum_bicolor_NCBIv3	ncRNAs<br>TEs
T. cacao	Theobroma_cacao_20110822	ncRNAs<br>TEs
T. aestivum	TGACv1	ncRNAs<br>TEs
T. urartu	ASM34745v1	ncRNAs<br>TEs
V. vinifera	IGGP_12x	ncRNAs<br>TEs
Z. mays	B73_RefGen_v4	ncRNAs<br>TEs
