# Supplementary material for: PlaNC-TE: a comprehensive knowledgebase of non-coding RNAs and transposable elements in plants
Source: Database (Oxford). 2018 Sep 13;2018:bay078. doi: 10.1093/database/bay078 (PMC6146122; doi:10.1093/database/bay078)
Supplement: Supplementary Table S2 [file bay078_tables2.pdf]

Table S2. TEs shared by Repbase and Ensembl Plants (EP) databases. Cutoff: 80% or higher identity and minimum of 80 nucleotides in the sequence.

Species	Repbase (TEs)	Repbase vs EP	**TEs %**
A. trichopoda	389	112	**28.79**
B. oleracea	23	20	**86.96**
C. crispus	1181	42	**3.56**
G. max	13	13	**100.00**
M. truncatula	211	200	**94.79**
M. acuminata	2	2	**100.00**
P. patens	56	29	**51.79**
P. trichocarpa	226	215	**95.13**
S. moellendorfii	84	76	**90.48**
V. vinifera	176	163	**92.61**
