# Supplementary material for: PlaNC-TE: a comprehensive knowledgebase of non-coding RNAs and transposable elements in plants
Source: Database (Oxford). 2018 Sep 13;2018:bay078. doi: 10.1093/database/bay078 (PMC6146122; doi:10.1093/database/bay078)
Supplement: Supplementary Table S3 [file bay078_tables3.pdf]

Table S3. ncRNA, TE and overlap entries.

Species	ncRNAs	TEs	ncRNA:TE Overlaps	No Overlap (ncRNA/TE)
Aegilops tauschii	2,345	2,253,478	276	2,100/2,253,218
Amborella trichopoda	728	153,241	219	519/153,032
Arabidopsis lyrata	833	116,145	466	471/115,729
Arabidopsis thaliana	1,429	43,442	722	770/42,728
Beta vulgaris	1,510	6,295	3	148/6,292
Brachypodium distachyon	1,278	106,890	180	1,124/10,510
Brassica napus	4,236	0	0	4,236/0
Brassica oleracea	1,469	160,486	381	1,113/160,125
Brassica rapa	1,291	97,576	483	845/97,107
Chlamydomonas reinhardtii	197	64,925	50	153/64,879
Chondrus crispus	16	910	2	14/908
Corchorus capsularis	1,216	252,864	546	687/252,347
Cucumis sativus	299	0	0	299/0
Cyanidioschyzon merolae	12	3,351	4	8/3,347
Dioscorea rotundata	154	0	0	154/0
Galdieria sulphuraria	13	109	1	12/108
Glycine max	426	373,988	0	426/373,988
Gossypium raimondii	511	341,493	0	511/341,493
Helianthus annuus	737	0	0	737/0
Hordeum vulgare	2,929	2,436,843	0	2,929/2,436,843
Leersia perrieri	899	112,058	220	694/111,838
Lupinus angustifolius	548	0	0	548/0
Manihot esculenta	472	0	0	472/0
Medicago truncatula	1,398	478,378	380	1,064/478,011
Musa acuminata	1,079	116,189	251	842/115,939
Nicotiana attenuata	375	0	0	375/0
Oryza barthii	948	185,442	161	795/185,281
Oryza brachyantha	443	380,576	245	280/380,333
Oryza glaberrima	701	695,468	528	366/695,066
Oryza glumaepatula	684	201,805	124	567/20,1681
Oryza indica	1,285	232,693	520	788/232,194
Oryza longistaminata	1,179	129,481	181	1,009/129,300
Oryza meridionalis	957	193,661	210	767/193,452
Oryza nivara	756	178,734	113	648/178,623
Oryza punctata	834	190,594	190	654/190,405
Oryza rufipogon	785	204,639	128	664/204,511
Oryza sativa	926	603,608	1,087	414/602,776
Ostreococcus lucimarinus	24	1,169	2	22/1,167
Phaseolus vulgaris	338	138	0	338/138
Physcomitrella patens	553	461,547	53	505/461,505
Populus trichocarpa	967	248,622	373	636/248,275
Prunus persica	234	29,599	0	234/29,599
Selaginella moellendorffii	1,312	116,938	532	840/116,434
Setaria italica	859	154,873	150	715/154,727
Solanum lycopersicum	1,404	575,496	295	1,138/575,203

Solanum tuberosum	1,409	993,218	395	1,065/992,827
Sorghum bicolor	1,359	428,546	338	1,034/428,210
Theobroma cacao	519	123,180	140	389/12,181
Trifolium pratense	1,236	0	0	1,236/0
Triticum aestivum	7,857	13,445,260	2,168	6,138/13,443,322
Triticum urartu	1,987	2,250,260	187	1,819/2,250,088
Vitis vinifera	637	281,476	131	521/281,349
Zea mays	1,797	1,791,946	1,915	794/1,790,828
**Total**	**58,390**	**31,217,630**	**14,350**	
