# Supplementary material for: PlaNC-TE: a comprehensive knowledgebase of non-coding RNAs and transposable elements in plants
Source: Database (Oxford). 2018 Sep 13;2018:bay078. doi: 10.1093/database/bay078 (PMC6146122; doi:10.1093/database/bay078)
Supplement: Supplementary Table S4 [file bay078_tables4.pdf]

Table S4. Overlapped records from PlanTE-MIR DB remapped in Ensembl Plants (EP) genomes.

Species	**PlanTE-MIR DB (Overlaps)**	EP (miRs)	EP (TEs)	**EP (Overlaps)**
A. trichopoda	**22**	21	22	**21**
B. distachyon	**2**	2	2	**2**
G. max	**4**	4	4	**4**
M. truncatula	**20**	20	20	**22**
O. sativa	**56**	56	57	**48**
P. patens	**1**	1	1	**1**
P. trichocarpa	**10**	10	10	**10**
S. bicolor	**35**	34	35	**33**
S. tuberosum	**1**	1	1	**2**
V. vinifera	**1**	1	1	**1**
