# Supplementary material for: PlaNC-TE: a comprehensive knowledgebase of non-coding RNAs and transposable elements in plants
Source: Database (Oxford). 2018 Sep 13;2018:bay078. doi: 10.1093/database/bay078 (PMC6146122; doi:10.1093/database/bay078)
Supplement: Supplementary Table S5 [file bay078_tables5.pdf]

1 –TSV custom file available on page “Download & JBrowse”. This file is composed to 24 columns which are filled with features about ncRNAs and TEs that overlap each other.

**ncRNAs features**	**TEs features**
Column 1: Region (Chr, Scaffold or Contig).	Column 13: Region (Chr, Scaffold or Contig).
Column 2: Source of the annotation.	Column 14: Source of the annotation.
Column 3: Biotype of the sequence.	Column 15: Type of the sequence.
Column 4: Start of the sequence.	Column 16: Start of the sequence.
Column 5: End of the sequence.	Column 17: End of the sequence.
Column 6: Length.	Column 18: Length.
Column 7: Minus or Plus.	Column 19: Minus or Plus.
Column 8: Score.	Column 20: Score.
Column 9: ID Gene.	Column 21: ID.
Column 10: Name.	Column 22: Repeat name.
Column 11: Description.	Column 23: Repeat type.
Column 12: Sequence.	Column 24: Sequence.
